# Misdiagnosis of Behcet’s disease lung involvement as *pulmonary cryptococcosis* on imaging: A case report

**DOI:** 10.1097/MD.0000000000050134

**Published:** 2026-08-14

**Authors:** Zhe Zhang, Zhi Yang, Haiyun Lin

**Affiliations:** a The Sixth Affiliated Hospital of Jinan University (Dongguan Eastern Central Hospital), Dongguan, Guangdong, China; d Department of General Practice, Shenzhen People’s Hospital, Shenzhen, Guangdong, China; b The Second Clinical Medical College, Jinan University, Shenzhen, Guangdong, China; c Department of Cardiology, The First Affiliated Hospital, Southern University of Science and Technology, Shenzhen, Guangdong, China

**Keywords:** Behçet’s disease, case report, imaging diagnosis, pulmonary cryptococcosis, pulmonary vasculitis

## Abstract

**Rationale:**

Behçet’s disease (BD) is a systemic vasculitis that rarely involves the lungs. Pulmonary manifestations of BD can radiographically mimic infectious pneumonia, posing a substantial diagnostic challenge and increasing the risk of inappropriate treatment. This case report describes a young female patient with BD who presented with pulmonary opacities initially misdiagnosed as cryptococcal pneumonia based on imaging findings.

**Patient concerns:**

A 28-year-old woman with a known history of BD presented with a 1-month history of fever, cough, and productive yellow sputum. She had been on long-term glucocorticoids, hydroxychloroquine, and colchicine for her underlying autoimmune disease.

**Diagnosis:**

Chest computed tomography revealed bilateral consolidation and nodules with subsequent cavitation, initially suggestive of pulmonary cryptococcosis. However, extensive microbiological workup, including next-generation sequencing of blood and bronchoalveolar lavage fluid, failed to identify any pathogen. Computed tomography pulmonary angiography demonstrated microthrombi and arteritis, leading to the final diagnosis of pulmonary vasculitis secondary to BD.

**Interventions:**

The patient was treated with methylprednisolone, which was gradually tapered, alongside supportive care. Antifungal and antibacterial therapies were discontinued after negative microbiological results were confirmed.

**Outcomes:**

The patient showed dramatic clinical improvement, with resolution of fever and respiratory symptoms. Follow-up computed tomography demonstrated significant regression of consolidation and complete resolution of cavitary lesions. At discharge, her laboratory parameters had normalized, and she remained stable during 1-year follow-up.

**Lessons:**

This case underscores that in BD patients, pulmonary opacities mimicking infection on imaging should not be automatically attributed to opportunistic pathogens. When empirical anti-infective therapy fails, and microbiological evidence remains negative within 72 hours, clinicians should promptly consider pulmonary vasculitis and pursue vascular imaging. Early recognition and timely initiation of immunosuppressive therapy can be lifesaving.

## 1. Introduction

Behçet’s disease (BD) is a **rare, systemic inflammatory disorder of unknown cause**, characterized by vasculitis with affecting both arteries and veins.^[1,2]^ It manifests with oral and genital ulcers, eye lesions, and skin involvement, and can also affect the nervous, gastrointestinal, and vascular systems.^[3,4]^ While BD can involve many organ systems, including the heart, kidneys, and lungs, pulmonary involvement is **exceptionally rare**, with pulmonary artery aneurysms occurring in only about **0.16% to 1.1%** of cases.^[5]^ Pulmonary involvement generally targets the pulmonary vasculature and parenchyma; symptoms typically include embolism, aneurysm, or autoimmune pneumonia.^[6,7]^ However, the varied radiographic signs and symptoms like fever, cough, and shortness of breath often lead to a misdiagnosis of infectious pneumonia.^[6,8,9]^

This article will present a case from our hospital with chest imaging initially suggestive of pulmonary cryptococcosis, which was ultimately diagnosed as pulmonary BD. This is the first report of such radiological features in an **East Asian patient**, providing a **crucial reference for accurately diagnosing similar cases.**

## 2. Case presentation

**Figure 1.**
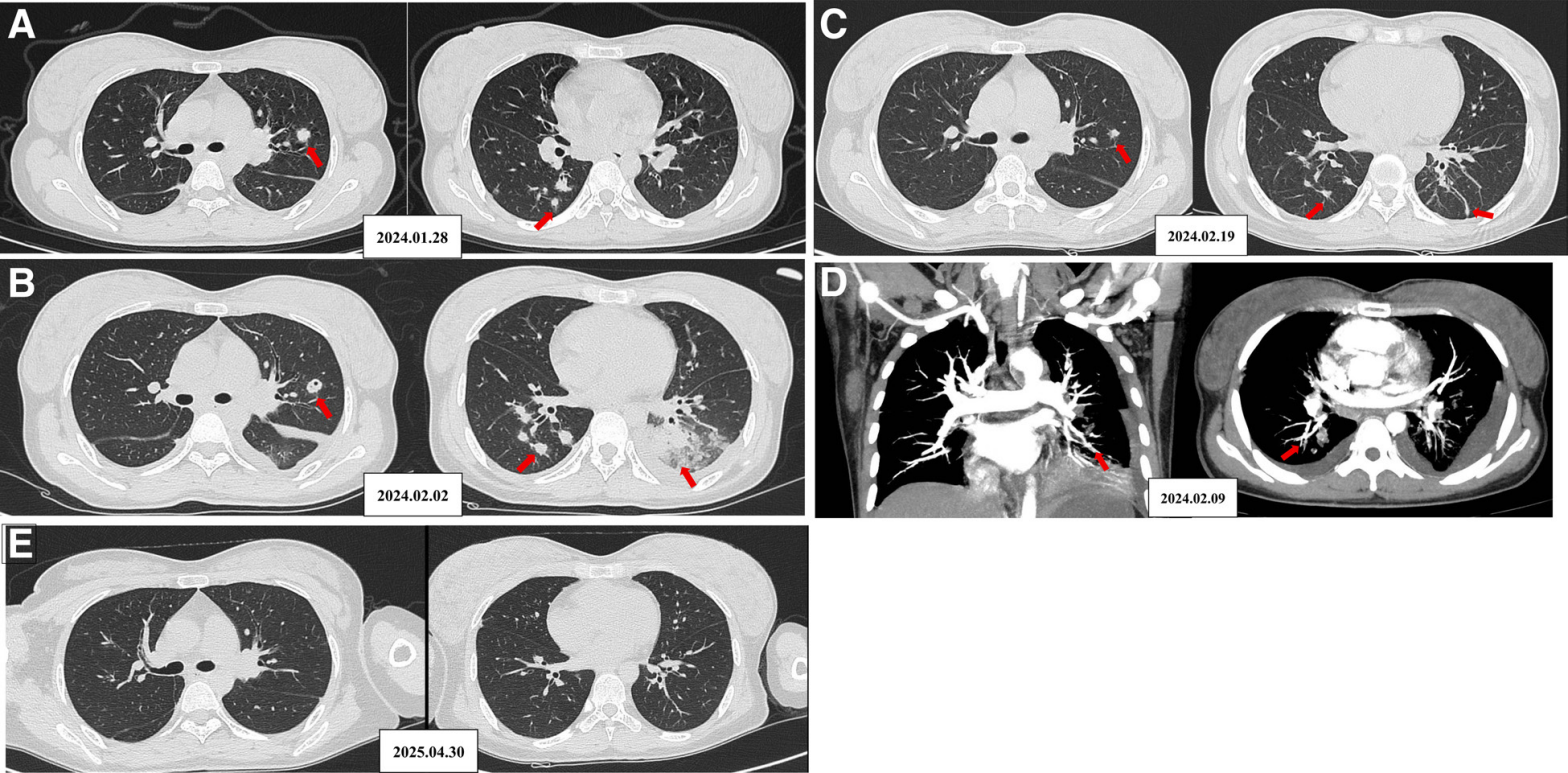
Temporal evolution of chest CT imaging findings. (A) shows large consolidation with peripheral inflammatory exudation in both lower lobes (January 28, 2024). (B) Left pneumonia progression more than before, with multiple cavitary lung nodules (February 2, 2024) (indicated by arrows). (C) Left lower lobe pneumonia was significantly absorbed more than (B) (February 19, 2024). (D) CTPA indicated microthrombi in the bilateral lower lobe pulmonary arteries (February 9, 2024) (indicated by arrows). (E) Follow-up chest CT on April 30, 2025, after regular outpatient treatment of the BD, showing near-complete resolution of the bilateral pulmonary opacities.

A 28-year-old woman was admitted with a month-long history of fever, cough, and **yellow sputum production**. Diagnosed with BD in June 2020, she was on glucocorticoids, hydroxychloroquine, and colchicine. No personal or family medical history was noted. Physical examination revealed bilateral bronchial breath sounds and **moist rales in the left hemithorax.** Initial labs showed a high WBC count of **18.74 × 10**^**9/L**^ and neutrophilia of **86.1%.** Elevated PCT and CRP levels were observed (Table 1). Chest computed tomography (CT) indicated new inflammatory changes and consolidation in the lower lung lobes of both lungs, with enlarged hilar and mediastinal lymph nodes (Fig. 1A). After treatment with moxifloxacin and a short course of dexamethasone a 2-day course of dexamethasone 5mg once daily, her inflammation markers improved, and she achieved normothermia.

**Table 1:** Laboratory indicators of the patients.

Date of examination	30/01	01/02	06/02	07/02	09/02	15/02	20/02
WBC (×10^9/L^; 4.0–10.0)	22.62	10.86	25.58	10.57	6.63	9.43	12.94
Neutrophils (%; 40–75)	83.80	74.50	90.20	88.50	85.90	67.00	73.60
CRP (mg/L, 0–8)	219.62	61.27	176.05	153.33	89.41	1.54	–
Hb (g/L)	91.00	91.00	85.00	77.00	83.00	93.00	92.00
PLT (×10^9/L^; 100–300)	338.00	393.00	337.00	325.00	423.00	528.00	410.00
PCT (ng/mL)	3.50	2.03	1.41	1.30	0.66	0.071	–
IL-6 (pg/mL)	114.20	20.09	487.60	3.04	1.70	3.69	–
Lactic acid (mmol/L; 0.7–2.1)	2.02	1.61	1.43	2.67	1.03	2.29	–

CRP = C-Reactive protein, PCT = procalcitonin, WBC = White Blood Cell.

However, she experienced a fever relapse on the third day, and a new CT scan showed worsening lesions with cavitary changes, hinting at *pulmonary cryptococcosis* (PC) (Fig. 1B). Despite a 3-day of fluconazole treatment, her fever persisted. Further tests revealed a WBC count of **25.58 × 10**^**9/L**^ and an absolute neutrophil count of **23.1 × 10**^**9/L**^. The antimicrobial strategy was revised to include voriconazole, and the antibacterial regimen was transitioned from moxifloxacin to voriconazole and piperacillin-tazobactam in response to the clinical and laboratory findings, but the patient’s fever symptoms have not improved. next-generation Ssequencing (NGS) (of both blood and bronchoalveolar lavage (BAL)) did not identify a bacterial pathogen (Table 2), but vascular ultrasonography and CT pulmonary angiography (CTPA) suggested segmental and subsegmental filling defects suggestive of microthrombi in the lungs (Fig. 1D). A multidisciplinary team (MDT) concluded her symptoms were due to BD-related vasculopathy.

**Table 2:** High-throughput sequencing report of pathogenic microbial nucleic acids in bronchoalveolar lavage fluid.

Bacteria	Fungus	Virus	Parasite	Drug resistance gene information	Virulence genes
Not found	Not found	Not found	Not found	Not found	Not found

In the Rheumatology and Immunology unit, she continued on piperacillin-tazobactam and began methylprednisolone, which was gradually reduced. She received supportive care, including antitussives, mucolytics, gastroprotective medications, iron supplementation, hematinic therapy, hepatoprotective agents, potassium repletion, and calcium supplementation. During this period, the patient did not experience any fever, and the respiratory symptoms, such as coughing and sputum production, were not present, indicating a stable clinical condition. The follow-up Complete Blood Count (CBC) showed a white blood cell (WBC) count of 12.94 × 10^9^/L, and an absolute neutrophil count of 9.5 × 10^9^/L. Additionally, there was a return to normal levels of inflammatory markers. A subsequent CT on February 19, 2024 displayed substantial absorption of the inflammatory opacities (Fig. 1C), and the cavities previously observed in some nodules had resolved.

Ultimately, she was discharged in a stable condition, with significantly improved laboratory results and a chest CT that demonstrated only residual consolidation and total resolution of the previously noted cavitations. Notably, a long-term follow-up CT **on April 30, 2025,** obtained after regular immunosuppressive therapy for the underlying BD, revealed near-complete resolution of the pulmonary parenchymal abnormalities (Fig. 1E).

## 3. Discussion

We present a unique case of pulmonary BD initially mimicking pulmonary Cryptococcosis (PC) on imaging, which is a rarely reported radiological diagnostic challenge in an East Asian population.

Pulmonary involvement in BD is rare, occurring in only 4% to 5% of cases, primarily manifesting as pulmonary artery aneurysms or vasculitis.^[5,6,9]^ Meanwhile, pulmonary cryptococcosis typically presents as solitary or multiple nodules with a predilection for the subpleural regions in immunocompromised hosts.^[10–12]^ In our patient, the underlying pathophysiology was BD-related vasculopathy. The inflammation of the vessel wall leads to increased permeability and microthrombi, resulting in pulmonary consolidation, nodules, and even cavitation.^[13,14]^ This contrasts sharply with PC, where the pathology involves granulomatous inflammation and necrosis caused by fungal invasion.

The imaging findings of nodules and cavitation initially favored a diagnosis of pulmonary cryptococcosis, especially given the patient’s long-term immunosuppressive therapy.^[8]^ However, several critical findings argued against infection. First, high-throughput sequencing (NGS) of both blood and bronchoalveolar lavage fluid (BALF) failed to detect any Cryptococcus DNA or other pathogens.^[15]^ Second, the lesions progressed rapidly despite adequate antifungal coverage with fluconazole followed by voriconazole. The clinical paradigm shifted when CTPA revealed microthrombi and arteritis. Crucially, the patient’s dramatic clinical and radiological response to methylprednisolone confirmed that the underlying etiology was autoimmune vasculitis rather than opportunistic infection.^[8,16]^ This was further corroborated by a 1-year follow-up CT, which showed near-complete resolution of all inflammatory opacities under regular BD-directed immunosuppressive therapy, with no antifungal agents administered during this period. Taken together, these findings establish that the pulmonary lesions were attributable to BD-related vasculopathy rather than cryptococcal infection.^[17]^ We emphasize that when empirical anti-infective treatment fails for >72 hours, and microbiological evidence remains negative, clinicians should strongly reconsider the differential diagnosis of noninfectious vasculitis, particularly in patients with underlying autoimmune diseases.^[18]^

We recognize that a lung biopsy was not obtained in this case. Nevertheless, considering the negative NGS results and the marked clinical improvement following corticosteroid administration, the diagnosis of BD-related vasculopathy is strongly supported. This case underscores a critical diagnostic principle: in BD patients with pulmonary opacities, such as nodules, cavitation, or consolidation, cryptococcosis should not be presumed solely on imaging.^[19]^ We propose a stepwise diagnostic algorithm: initiate empirical broad-spectrum anti-infectives while awaiting rapid microbiological assays (NGS and serum cryptococcal antigen); if no pathogen is identified and clinical deterioration persists beyond 72 hours, promptly pursue vascular imaging (CTPA) to assess for underlying vasculitis or thrombosis; and timely initiation of immunosuppressive therapy can be lifesaving. Clinicians and radiologists must recognize that in BD, “infection-mimicking” radiographic patterns often reflect active vasculitis rather than superimposed infection.

## Acknowledgments

The authors wish to acknowledge the AI agent for linguistic refinement.

## Author contributions

**Conceptualization:** HaiYun Lin.

**Writing – original draft:** Zhe Zhang.

**Writing – review & editing:** Zhi Yang, HaiYun Lin.
